# Narrative Review: Pyoderma Gangrenosum

**DOI:** 10.7759/cureus.51805

**Published:** 2024-01-07

**Authors:** Ann N Park, Aishwarya Raj, Joe Bajda, Vasavi R Gorantla

**Affiliations:** 1 Anatomical Sciences, St. George's University School of Medicine, True Blue, GRD; 2 Biomedical Sciences, West Virginia School of Osteopathic Medicine, Lewisburg, USA

**Keywords:** pyoderma gangrenosum, skin ulcer, neutrophilic dermatosis, post-operative pyoderma gangrenosum, vegetative pyoderma gangrenosum, peristomal pyoderma gangrenosum, autoimmune-mediated inflammatory dermatosis, ulcerative pyoderma gangrenosum, complications of inflammatory bowel disease

## Abstract

Pyoderma gangrenosum (PG) is a skin lesion, characteristically a neutrophilic dermatosis, that can be complicated by rapid progression, necrosis, and ulceration. This is an important pathology to be discussed given that there are no established criteria for diagnosis or treatment. This review aims to elucidate characteristics and variations of PG that distinguish it from other ulcerative skin lesions. Variability in presentation can lead to missed or incorrect diagnosis, and some of the currently proposed criteria for categorizing and diagnosing PG have been included here. These criteria distinguish PG in terms of the nature of the lesion, the location, etiology, responsiveness to immunosuppressive therapy, and patient history. The etiology and pathogenesis of PG remain unknown, but we summarize prominent theories and explanations. Furthermore, recent research indicates that the incidence of PG has a strong correlation with autoimmune conditions, particularly inflammatory bowel disease. Major treatments for PG coincide with these findings, as the majority involve targeted anti-inflammatories, immunosuppressants, and surgical interventions. These treatments are addressed in this review, with added context for local versus systemic disease.

## Introduction and background

Although the etiology of pyoderma gangrenosum (PG) is unknown, it is largely believed to be an inflammatory complication of autoimmune origin [1,2]. Considered a neutrophilic dermatosis, misdiagnosis remains common because of PG’s similarities to other skin lesions and a lack of specific clinical tests for diagnosis [3-10]. It was first described as a distinct condition termed “phagédenisme géométrique” in 1908 by a French physician, Louis Brocq [11]. Brocq described patients who presented with ulcerations consisting of three distinct characteristics: a ridge, an external slope of the border, and an internal slope of the border. He characterized the internal slope as erythematous, and the external slope as sharp as a cliff with dimpled purulent cavities [7,11]. Originally thought to be a dissemination of bacterial infection caused by *staphylococci* or *streptococci* species, the etiology of PG remains elusive to date [3,7,8,11,12].

The term “pyoderma gangrenosum” was assigned in 1930 by Brunsting who described a series of patients affected by either ulcerative colitis or idiopathic chronic purulent pleurisy [13]. Pyoderma is used to describe the purulent nature of the infection, while gangrenosum is indicative of the progressive necrotic nature of the condition. Brunsting believed that the origin of disease was infectious in nature, likely due to pyogenic organisms. Trends emerging throughout the 1940s suggested that PG was highly associated with ulcerative colitis but also presented in conjunction with a myriad of conditions such as autoimmunity, infection, or trauma, to name a few [3,7,8,12,14-21].

PG most commonly affects adults between 40 and 60 years of age, often in the setting of pre-existing autoimmune pathology [8,22]. PG typically begins as a tender nodule or plaque that progresses, erupts, and ulcerates, with the lower extremities being the most commonly affected area. The ulcer becomes sharply marginated and is surrounded by an erythematous border. In accordance with its name, the underlying tissue becomes necrotic, including the skin, subcutis, and, at times, extending to the muscle. Lesions are often multiple and recurrent, with purulence and hemorrhage, which can ultimately heal as atrophic scars [3].

## Review

Pathophysiology

**Figure 1:**
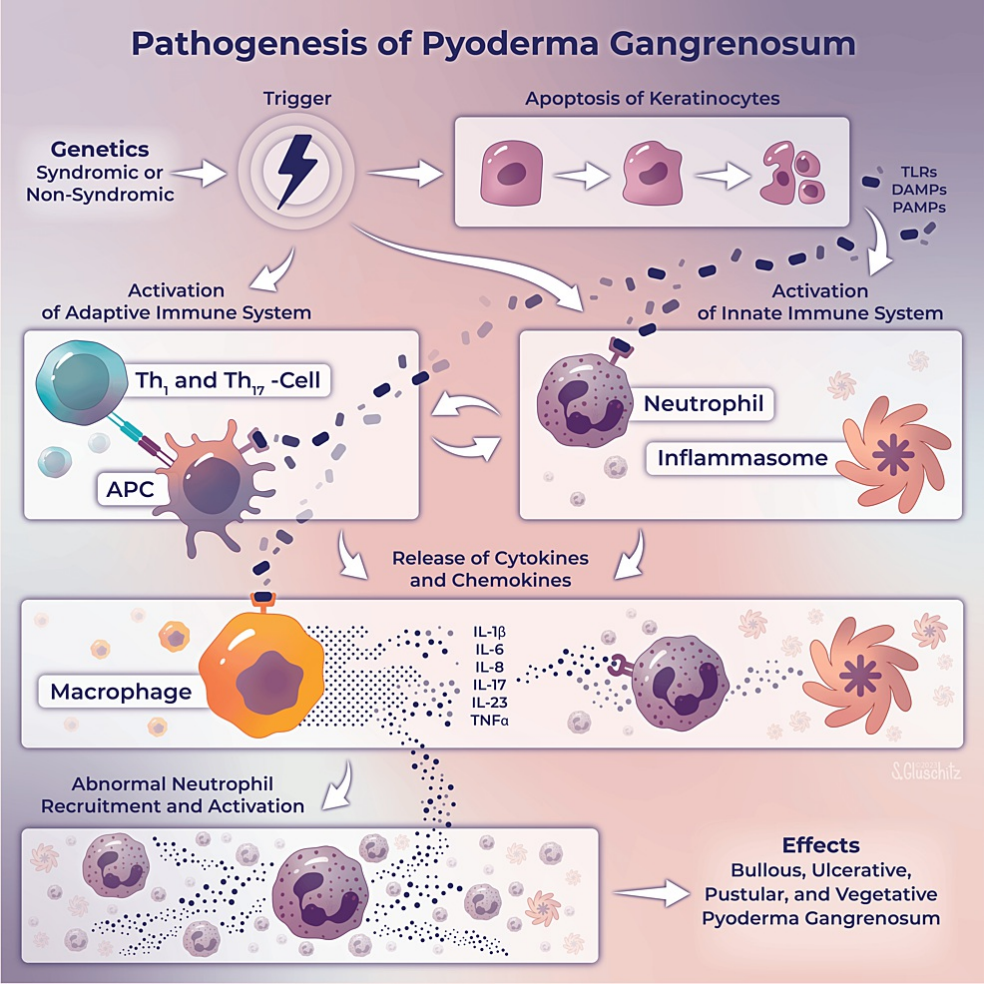
A proposed pathogenesis of pyoderma gangrenosum Printed with permission from Sarah Gluschitz, MA, CMI © CC-BY-ND 2023. Based on previously published material by Hobbs and Ortega-Loayza, with permission [7].

Early hypotheses of PG etiology included infection, circulating autoantibodies, or the Shwartzman reaction, a thrombohemorrhagic skin reaction associated with disseminated intravascular coagulopathy [23]. The condition is now largely thought to be due to autoimmune dysregulation given that immunosuppressants, often in combination, can facilitate healing and remission [17]. Dysregulation of innate immunity, specifically in neutrophil chemotaxis, is thought to be one of the underlying mechanisms in the pathogenesis of PG [3]. As the term neutrophilic dermatosis implies, PG biopsies show abundant mature neutrophils within the dermis. Dysregulated β2-integrin (CD18) signaling, involving complement receptors (CR) 3 and 4, have been experimentally demonstrated to play a role in the pathology, with anti-neutrophilic agents such as colchicine and dapsone having moderate effectiveness against progression of PG. More broadly, PG is characterized by abnormalities in cytokine signaling, immune regulation, and neutrophils, manifesting in genetically predisposed individuals. Upregulation of neutrophil chemotactic factors such as tumor necrosis factor alpha (TNFα), interleukin (IL)-1β, IL-6, IL-8, IL-17, and IL-23 have also been found [24,25]. This helps explain why many of the affected individuals have underlying conditions such as irritable bowel disease, PAPA (pyogenic arthritis, PG, and acne) syndrome, and many other inflammatory pathologies [14,26]. T-cells are thought to play a role in the immune-mediated destructive pattern, particularly at the wound margins [8,23]. An estimated 25-50% of PG is considered of idiopathic origin, but associations with systemic autoimmune conditions such as inflammatory bowel disease (IBD) suggest that the rapidity of progression is driven by an exaggerated immune response [19]. An outline of the proposed pathogenesis is shown in Figure 1.

Neutrophilic dermatoses can be thought of as a spectrum of polygenetic dermatological conditions of which PG is a member. There is also a known group of monogenic autoinflammatory syndromes that can present with lesions resembling the classical ulcerative PG, but these syndromes are rare and usually arise in childhood or early adulthood [27]. Gene mutations such as *PSTPIP1* and *MEFV* have also been found in patients with neutrophilic dermatoses, suggesting that a spectrum of genetic changes contributes to the development of PG [8].

Presentation and course

PG is strongly associated with autoimmunity or systemic disease, namely IBD or hematologic malignancy [22,28,29]. The incidence is higher in patients aged 40-60 years [8,22]. However, PG can present at any age, starting from infancy [30,31].

**Figure 2:**
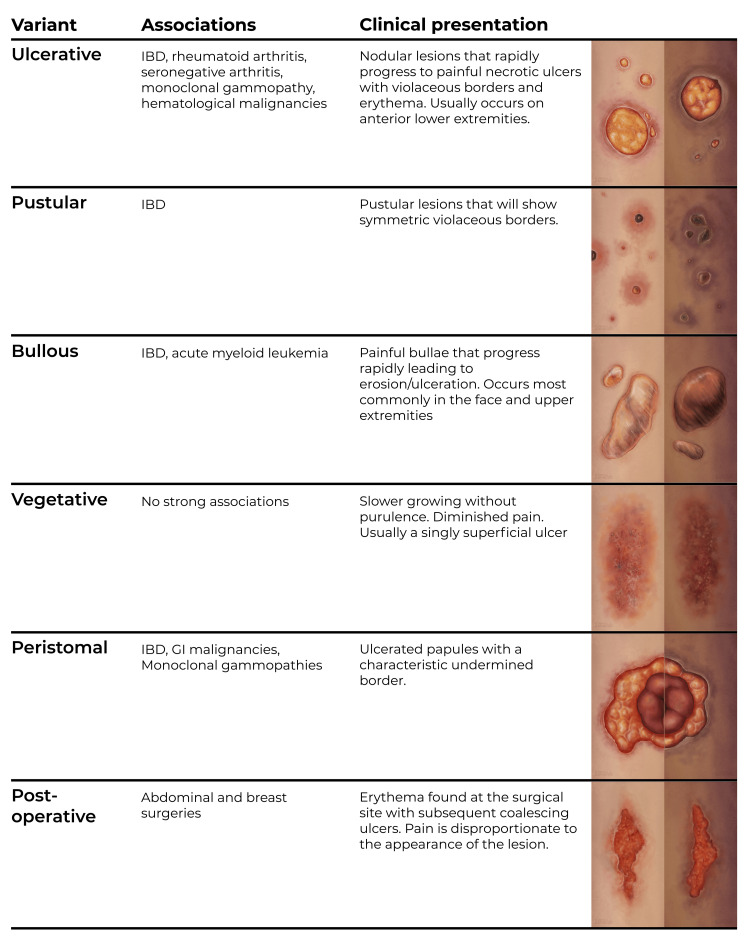
Variants of pyoderma gangrenosum Printed with permission from Sarah Gluschitz, MA, CMI © CC-BY-ND 2023. Vegetative lesion based on previously published material by Bhat, with permission [33].

Patients typically present with sterile pustules that proceed to ulcerate and become painful with violaceous borders. Lesions commonly present in the pretibial area. Often, the borders may be undermined (meaning worn and damaged) with surrounding erythema. PG can progress rapidly, and this makes swift identification extremely important. Systemic manifestations include fever, malaise, weight loss, and myalgia [21]. The course of the disease varies from mild to relapsing, with a high associated morbidity. PG skin lesions have been classified into several categories: ulcerative, pustular, bullous, vegetative, peristomal, and postoperative [8,9,32]. Figure 2 presents these variants in detail, including illustrations for both light and dark skin.

The ulcerative form is also known as classical PG and comprises approximately 85% of cases [26]. Gameiro et al. described two distinct stages of the classic ulcerative form: active ulcerative stage and wound healing stage [15]. The ulcerative stage is characterized by a peripheral inflammatory halo with raised erythematous, sometimes necrotic, edges that rapidly expand. Extension of the ulcer edge can be associated with the development of surrounding pustules. The wound healing stage demonstrates Gulliver’s sign: string-like projections of the epithelium that bridge the border of the ulcer and surrounding normal skin. Ultimately, this results in an atrophic cribriform scar that resembles “cigarette paper” [15,21].

Pathergy, an exacerbation of skin injury due to manipulation of the affected tissue, is a common complication with PG lesions. This complication particularly aids in the development of peristomal PG lesions [15]. Age typically does not alter clinical presentation, with the exception of pathergy-related cases. Patients older than 65 years had higher rates of pathergy compared to those younger (36.3% vs 24.3%; P=0.02) [34].

Manifestations of PG most commonly occur on the lower extremities and the trunk, with rare cases manifesting elsewhere throughout the body. Bissonnette et al. conducted a systematic review of these rare PG infections involving the oral cavity, finding a total of 20 cases in English and French literature. The oral mucosa can undergo significant trauma through mastication, brushing teeth, or dental procedures, and yet the number of reported cases of PG remains extremely low. Bissonnette et al. suggested that this may be due to incorrect or absent diagnoses [5].

Garg et al. examined an uncommon case of penile ulcer in a 50-year-old diabetic male. The patient presented with two ulcers over the glans penis, minimally painful but ulcerated and rapidly increasing in size. Extensive laboratory investigations were negative for sexually and non-sexually transmitted infections, vasculitis, and malignancy. Biopsy of the affected area revealed a dense neutrophilic infiltrate of the dermis with lymphocytes and plasma cells. Diagnosis of PG was made out of exclusion from other causes. The patient showed rapid improvement with colchicine, dapsone, and prednisolone [35].

Due to the unpredictable nature of PG, incredibly rare extracutaneous manifestations have been observed, and include, but are not limited to, the bones, eyes, lungs, spleen, and central nervous system [15,36]. Extracutaneous lesions tend to be misdiagnosed for an infectious cause, as the infiltrates are not temporally related to typical cutaneous lesions of PG. A case study reported by Santa Lucia et al. examined a 56-year-old woman already diagnosed with cutaneous PG maintained on immunosuppressive therapy, returning to the hospital multiple times with pulmonary, pericardial, hepatic, splenic, and pancreatic involvement [37]. Suspicion of infection directed the internal medicine team to discontinue immunosuppressants and start the patient on broad-spectrum antibiotics and antifungals. As the patient’s condition continued to worsen, extracutaneous PG was considered due to the concurrent flare-up of the patient’s cutaneous lesions. The patient was restarted on higher doses of immunosuppressants, leading to resolution of active lesions. As evidenced by this case, extracutaneous manifestations must be considered and investigated for all patients presenting with previously diagnosed cutaneous PG.

Diagnosis

For most of its historical course, the diagnosis of PG was one of exclusion, called a "great imitator," and true incidence is debatable as skin ulcerations are often misdiagnosed as PG [14,38,39]. Moreover, diagnosis can be delayed due to a lack of distinguishing autoantibodies, complement changes, immune complex circulation, or human leukocyte antigen (HLA) associations [19]. Non-specific findings include elevated erythrocyte sedimentation rate and C-reactive protein, as well as leukocytosis [40]. The presence of autoantibodies rules out differential diagnoses for chronic non-healing wounds associated with systemic lupus erythematosus, scleroderma, Sjogren's syndrome, rheumatoid arthritis, vasculitides, anti-phospholipid syndrome, genetic prothrombotic state, or infection (mycobacterium tuberculosis, human immunodeficiency virus [HIV], hepatitis B virus, hepatitis C virus) [41]. Similarly, biopsy is an exclusionary tool in ruling out alternative etiologies. The biopsy should demonstrate dermal edema and neutrophilic infiltration and ideally includes the ulcer edge [42]. An important clue is the rapidity at which PG progresses, i.e., a 1-2 cm/day expansion of wound margin or greater than 50% expansion over a month [43,44]. Most decisions are made clinically after a lack of response to antibiotics and after a positive response to immunosuppressive therapies.

Diagnostic Criteria for PG

There have been a handful of proposed diagnostic models for PG, namely for the most common ulcerative types. Su et al. suggested two major criteria and two minor criteria based on common features of ulcerative PG derived from a retrospective study of medical records (Table 1) [44]. These features are not unique to PG and can lead to misdiagnosis when histopathological or clinical features are too heavily weighed. Moreover, this criterion maintains the nature of a diagnosis of exclusion, which is exhaustive and unrealistic in practice.

**Table 1:** Criteria for ulcerative pyoderma gangrenosum Early diagnostic criteria proposed by Su et al. [6,44].

Major criteria	Minor criteria
Necrotic ulcer with the following: preceding lesion of papular, pustular, or bulla-like quality, pain out of proportion to ulcer size, rapid progression (1-2 cm/day or 50% enlargement in <1 month), or border that is irregular, violaceous, and undermined	History of pathergy or presence of cribriform scarring
	Co-existence of PG-associated disease, e.g., inflammatory bowel disease, malignancy
Exclusion of other causes of cutaneous ulcer (via historical data, biopsy, and laboratory investigation)	Biopsy showing sterile neutrophilic infiltration, with or without mixed inflammation or lymphocytic vasculitis
	Swift (<1 month) response to systemic corticosteroids (1-2 mg/kg/day)

PG can be mistaken for chronic venous ulcers. One distinguishing characteristic is that PG tends to present on both upper and lower legs, whereas chronic venous ulcers are typically restricted to lower legs and feet. In addition, patients with PG can present with purulent discharge and pustule formation, features that are not found in patients with chronic venous ulcers. Because PG bears a presentation similar to venous ulcers, a retrospective study comparing the two conditions led to PARACELSUS, an acronym for the 10-criterion comprising a point-based diagnostic tool for PG (Table 2) [43,45].

**Table 2:** PARACELSUS criteria Criteria used for differentiating pyoderma gangrenosum from venous ulcers [6,7].

Criteria	Clinical features
P - Progressive disease	Rapidly progressing ulcer (<6 weeks)
A - Assessment	Absence of alternative relevant differentials
R - Reddish	Violaceous wound margins
A - Amelioration	Alleviation by immunosuppressant drugs
C - Characteristics	Characteristic irregularly shaped ulcer
E - Extreme Pain	Ulcer extremely painful for the patient (>4/10, visual analog scale)
L - Localization	Lesion exhibits pathergy; lesion location is restricted to the site of trauma
S - Suppurative	Suppurative inflammation found on biopsy and histology
U - Undermined	Undermined wound border
S - Systemic	Presence of associated systemic disease

The most current recommended guidelines for diagnosis of ulcerative PG are based on consensus criteria established by the Delphi exercise, as described by Maverakis et al. The major criterion is evidence of neutrophilic infiltrate along the ulcerative border, as well as eight additional minor criteria including exclusion of infection, pathergy, history of IBD or inflammatory arthritis, history of papule, pustule, or vesicle that ulcerates within 4 days of initial appearance, peripheral erythema and undermined border, multiple ulcerations with at least one on the lower extremity, cribriform scar, and responsiveness to immunosuppressive therapy (Table 3) [42].

**Table 3:** Delphi exercise criteria A more recently proposed diagnostic criteria for pyoderma gangrenosum [6,7]. IBD, inflammatory bowel disease

Major criteria	Minor criteria
Neutrophilic infiltrate along ulcerative borders	No evidence of infection
	Pathergy
	History of IBD or inflammatory arthritis
	History of papule, pustule, or vesicle that ulcerates within 4 days of initial appearance
	Peripheral erythema and undermined border
	Multiple ulcerations with at least one on the lower extremity
	Cribriform scar following wound healing
	Responsiveness (shrinking of wound) to immunosuppressive therapy

Although these proposed methods can help form a basis for PG as a differential, it can be useful to maintain it as a placeholder as alternative diagnoses are explored. Broadly, the alternatives encompass vasculitides, vascular occlusive disease, cancer, infectious disease, drug reactions, and exogenous tissue injury [9]. Table 4 lists the wide spectrum of conditions previously misdiagnosed as PG.

**Table 4:** Differential diagnosis for PG A broad list of differential diagnoses should be considered against PG [3,5,9,12,15,16,19,32,39,46,47]. PG, pyoderma gangrenosum

Differential diagnosis	Characteristics
Vasculitis	Pathologies such as granulomatosis with polyangiitis, polyarteritis nodosa, Takayasu vasculitis, cryoglobulinemic vasculitis, anti-phospholipid syndrome, or Behcet's disease can present with similarities to PG.
Vascular/venous occlusive disease	Conditions such as calciphylaxis and venous ulcers can present with rapidly progressive and painful skin lesions.
Cancer	Patients with leukemias and lymphomas can present with skin ulcers that resemble PG. Examples include T-cell lymphoma, mycosis fungoides, and leukemia cutis.
Infectious disease	Certain infections, such as sporotrichosis, can resemble PG due to rapidly progressive ulcerative lesions that are often extremely painful. Cellulitis and necrotizing fasciitis have also been seen to bear some resemblance to lesions caused by PG. Mycobacterium, fungal, viral, leishmaniasis, and amebiasis can also be infectious agents associated with PG-like lesions.
Exogenous tissue injury	Necrotizing skin ulcerations can occur due to insect or spider bites and mimic features of PG.
Drug reaction	Drugs that cause pustular lesions can resemble the pustular version of PG, bromoderma, drug-induced lupus, hydroxyurea-induced ulcerations, or injection drug abuse with secondary infection.
Autoimmune	Systemic lupus erythematosus, rheumatoid arthritis, cutaneous Crohn’s disease

Other Considerations

More than 70% of patients with PG have comorbidities [30,35-36]. The most frequent comorbidity is IBD, with ulcerative colitis (33%) being more frequently associated than Crohn’s disease (7%) [48]. Kridin et al. found a 15-fold increase in odds of PG for individuals with ulcerative colitis, and more so within a year of diagnosis [49]. That being said, the relative risk of PG after an initial diagnosis of UC is unknown. Other commonly associated diseases include arthritis, monoclonal gammopathies, and hematologic malignancies, to name a few [19]. PG as a primary diagnosis (idiopathic PG) is reported in 22% or even more than 40-50% of patients, suggesting a complex relationship between PG and other disorders of immune dysfunction [19,50]. Studies also suggest ethnic differences in PG associations; hematologic malignancies accounted for the most common comorbidity (24.5%) before IBD (18.9%) in Korean patients, and up to 30% of Takayasu disease patients in Japan were associated with PG [19,51].

Patients can also present with mixed etiologies, with PG arising in the setting of ongoing chronic venous and/or arterial insufficiency. A case report by Wong et al. described a 60-year-old female with a 40-pack-year smoking history who presented with two right lower extremity ulcerations for a six-week period, which began as small brown bumps over the anterior shin that quickly progressed to painful ulcerative lesions with necrotic eschars. The right leg also demonstrated mild edema and non-palpable pulses. Surgical debridement procedures (three in total) were performed, and the patient was given IV antibiotics. The patient’s wounds and pain worsened severely postoperatively. Tissue biopsy after the fourth debridement procedure led to a diagnosis of PG. As demonstrated by this case, PG in the setting of overlapping etiologies makes diagnosis particularly challenging [10].

Although the incidence of PG is relatively rare, the morbidity and mortality associated with misdiagnosis make PG an important differential. PG may get overlooked partly because it lacks adequate coverage in medical resources. Erickson et al. investigated the coverage of PG in medical education by sampling several major resources commonly used in family medicine, internal medicine, and infectious disease. Nine of 22 journals and 12 of 15 textbooks mentioned PG. None included PG as a differential diagnosis within necrotizing and soft tissue infections or venous ulcers. Because PG often mimics these conditions, including PG as a differential may reduce the rates of misdiagnosis [52].

Potential Aids in Assisting PG Diagnosis

Bacterial fluorescent imaging is a bedside tool that uses 405 nm light to predict the bacterial burden of cutaneous wounds. It reportedly has a positive predictive value of 95% for common pathogens associated with chronic wounds such as *pseudomonas*, *klebsiella*, *staphylococcus*, and *proteus* species, with diagnostic accuracy of infection 2.2-fold greater than diagnosis of infection based on clinical signs and symptoms alone [53]. MacLeod et al. describe an interesting case report of a groin and thigh wound with differential diagnosis including both soft tissue infection and PG. Bedside fluorescent imaging showed little bacterial burden and, in combination with histopathology and wound culture results, guided the ultimate diagnosis and treatment of PG, sparing the patient of potentially detrimental debridement procedure [54].

Birkner et al. offered artificial intelligence as a potential diagnostic tool based on the analysis of wound photographs. In this study, a deep convolutional neural network (CNN) was trained to analyze wound photographs of PG compared with other chronic leg ulcers. The CNN’s ability to identify PG in 36 of 69 ulcer photographs was compared to that of a panel of 18 dermatologists with respect to sensitivity, specificity, and accuracy. CNN had 97% sensitivity (95% CI: 84.2-99.9%) in comparison to dermatologists at 72.7% (95% CI: 54.4-86.7%; P<0.03), though dermatologists’ specificity was slightly superior (88.9% vs 83.3%) [55].

Genotyping may have a potential role, albeit in the distant future, in informing some of the pathogenic mechanisms behind PG. PGAAIS (pyoderma gangrenosum-associated autoinflammatory syndromes) has been described as a distinct clinical class sharing inherited mutations in several genes, notably *PSTPIP1* [56]. Conditions included in this class are PAPA (pyogenic arthritis, pyoderma gangrenosum, and acne conglobata), PASH (pyoderma gangrenosum, acne, and suppurative hidradenitis), and PAMI (PSTPIP1-associated myeloid-related proteinemia inflammatory syndrome). Saternus et al. described a four-step algorithm in identifying autoinflammatory syndromes based on an initial diagnosis of PG [57]. As molecular diagnostics are increasingly available and affordable, genotyping can become another tool in elucidating such syndromic relationships.

Treatment and management

Approach to Treatment

 Treatment delay significantly impairs the outcome and resolution of the wounds; therefore, timely diagnosis and initiation of therapy are of paramount importance in managing PG. Little data exist on successful interventions in PG, such that there is no “gold standard” guideline for treatment. Treatment is broadly grouped into three categories including, topical, systemic, and surgical treatments. Treatment regimens tend to be dictated by the severity of the ulcers. Mild disease can be cared for with local wound care and topical or intralesional therapies. Systemic therapy is considered for disease that is widespread, rapidly progressing, or failed containment by local agents. Corticosteroids and immunosuppressive drugs such as cyclosporine have been reportedly the most successful pharmacotherapies [9,16]. Biologic agents, such as infliximab, are on the rise [26]. Surgical intervention has largely been controversial due to the increasing potential of pathergy. However, certain surgical methods have shown some effectiveness in reducing wound size, especially when combined with a pharmacological approach. Pain is majorly associated with PG and should be addressed with controlled analgesics that may be tapered as the wound begins to heal [15].

Local Therapies

The primary principle in the treatment of milder disease forms begins with gentle wound care. Moisture-retentive occlusive dressings can be used for chronic wounds to promote re-epithelialization, angiogenesis, and collagen synthesis. These dressings, however, would be contraindicated for exudative and purulent lesions. Absorptive dressings would be preferred for such lesions. Compression is indicated where there is associated edema. Topical antibiotics may be used with caution, but not as empiric therapy, to prevent resistance and delayed wound healing [3].

Topical corticosteroids appear to be the first-line therapy for PG. Corticosteroids inhibit pro-inflammatory mediators, which are responsible for the progression of the disease. Class I corticosteroids are typically used, but weaker classes, such as methylprednisolone, have been used as adjunctive therapy with silver sulfadiazine. Calcineurin inhibitors such as cyclosporine and tacrolimus are also popular choices for topical therapy. Inhibition of calcineurin ultimately results in the inhibition of T-cell activation and proliferation. Both cyclosporine and tacrolimus have been proven effective as monotherapy, but tacrolimus has shown greater potency than cyclosporine in vitro [58]. A larger wound, however, carries a greater risk that a drug is systemically absorbed and causes adverse effects. Pimecrolimus, another calcineurin inhibitor, has lower bioavailability comparatively and thus may be considered in reducing systemic effects [59].

Intralesional injections of corticosteroids can be useful as adjunctive therapy. The intralesional route can achieve a wider spread effect and avoid systemic adverse effects at the same time. However, it should be used with caution to avoid pathergy and disrupt wound healing [3]. Intralesional triamcinolone, dexamethasone, and methotrexate have also shown some clinical effects [59]. Protein C and Timolol gel, which are used for chronic venous and diabetic ulcers, show some promise in future treatment of PG [15].

Systemic Therapies

Topical treatment is insufficient for progressing disease, and, thus, a systemic approach should be considered. The mainstay treatment remains oral corticosteroids to slow disease progression. Cyclosporine can be used as a steroid-sparing approach either alone or in combination with oral corticosteroids [43]. Ormerod et al. conducted a randomized controlled trial (n=112) comparing the efficacy of corticosteroids versus cyclosporine individually in terms of rapidity of wound healing. This study showed no significant difference between oral prednisolone 0.75 mg/kg/day and 4 mg/kg/day of cyclosporine, with 47% of ulcers being healed at six months in both cohorts. Frequency of adverse reactions was similar in both groups, but prednisolone was found to have a higher likelihood of severe reaction [41].

Mycophenolate mofetil inhibits purine and pyrimidine synthesis through the inhibition of inosine-5'-monophosphate (IMP) dehydrogenase and consequently interferes with T- and B-cell proliferation. Cases have been reported where mycophenolate mofetil monotherapy has led to complete remission of PG [12].

Azathioprine disrupts purine synthesis and can impair T-cell activation. It has been used as a steroid-sparing agent and is particularly useful as an adjuvant in patients with associated IBD. Thalidomide has shown effectiveness in refractory PG by its modulation of NF-κB related cytokines and chemokines. It can be used alone or in combination with corticosteroids [17].

The anti-inflammatory activity of intravenous immunoglobulin (IVIG) allows it to be an alternative systemic treatment that appears helpful in preventing superinfection of the ulcers. Solitary lesions are found to be more responsive to IVIG regardless of location [43].

Biologic Therapies

There is emerging evidence on the use of biologic agents in the treatment of PG. Infliximab is the most popular choice for biological agents in the treatment of PG. Infliximab is a chimeric inhibitor of TNF⍺, a cytokine with a key role in inflammation. Brooklyn et al. conducted a randomized controlled trial in which 69% of patients had positive clinical outcomes with a single dose (5mg/kg) of infliximab [32]. Adalimumab, a fully human recombinant monoclonal antibody that inhibits TNF⍺ from binding to its receptor, was found to be effective as a second-line therapy in patients refractory to first-line infliximab [60].

Multiple interleukin inhibitors are also considered effective in the treatment of PG. A semi-systematic review compiled the data that showed a complete response rate of 31% with anakinra, an IL-1 antagonist, and 71% with ustekinumab [49]. Ustekinumab binds the shared p40 subunit of IL-23 and IL-12. IL-23 is the driving force for the T-helper cell conversion to Th17, which is significantly elevated in PG lesions and associated with low levels of regulatory T-cells [15].

Surgical Therapies

Initial surgical debridement is controversial in PG treatment as it can lead to pathergy, as mentioned earlier, an exacerbation of skin injury after manipulation of the primary lesion [16].

Hyperbaric oxygen therapy (HBOT) is well documented as an adjuvant therapy for PG. It provides symptomatic relief and aids in wound healing. Araujo et al. reported a case in which HBOT was used in the treatment of a deep, extensive ulcer exposing muscular tissue with areas of pus and necrosis [20]. Granular tissue began to form after 45 days of treatment with corticosteroids, azathioprine, and HBOT. Partial autologous skin graft was successful after five months with concurrent azathioprine and HBOT. There was no recurrence or complications after 10 months.

Skin grafts can be an effective treatment for destructive ulcerations found in PG but are often avoided due to concern of surgery-induced pathergy. Morgenstjerne-Schwenck et al. conducted a systematic review of skin grafting procedures for vasculitic ulcers and PG. For example, one study reported that 76.5% of PG that underwent skin grafting procedures achieved complete healing, with a mean healing time of 11 weeks [61].

Negative pressure wound therapy (NPWT) has been considered a useful method to promote the formation of granulation tissue and aid in the wound healing process. Almeida et al. performed this procedure for seven days and followed with a full-thickness skin graft that had satisfactory integration. Three months later, the patient was given an increase in cyclosporine and corticosteroid therapy, after which the lesion completely regressed and remained without recurrence at the 18-month follow-up [62]. Eisendle et al. reported that NPWT increases skin graft take with split-thickness skin grafting and suggested that immunosuppressive therapy should be implemented post-surgically to prevent recurrence [63].

Chan et al. reported a case in which a PG lesion secondary to ulcerative colitis was treated with a fetal bovine dermis (FBD) graft [64]. The FBD was bolstered with nonadherent cellulose acetate mesh and wrapped with a zinc oxide compression boot. After four days, the dressing was removed, and the wound was noticeably less hyperemic, and the graft was successfully taken. The patient was discharged on a prednisone taper. Triamcinolone acetonide was injected to the periphery of the wound. The wound bed showed near-complete response at the one-month follow-up. Authors concluded that surgical intervention combined with immunosuppressive therapy can aid in wound healing and should be considered for extensive wounds [64].

## Conclusions

PG remains difficult to diagnose and treat. Although the etiology remains unknown, current proposals involve upregulation of neutrophil chemotactic cytokines, with subsequent inflammatory response. Furthermore, there is some evidence of polygenic predispositions for individuals most susceptible to PG. The rapidity of its progression and the potentially fatal effects of systemic disease make its early detection of utmost importance. PG is often a diagnosis of exclusion. Variability of presentation and histopathology can complicate diagnosis. Moreover, ulcerative lesions can present similar to conditions such as chronic venous insufficiency, hence delaying appropriate treatment. Treatment depends on the nature of the lesion and whether there is systemic or localized involvement. Corticosteroids and immunosuppressive therapies are currently the preferred treatments, with the most success achieved in cases of early detection and treatment. Due to the inflammatory nature of PG, cytokine inhibitors have also shown efficacy in controlling immune-mediated damage. Moreover, surgery may be necessary and should be considered in combination with immunotherapy for certain individuals. Refinement of the etiology and pathophysiology, with identification of possible genetic predispositions, can lead to earlier diagnosis and possibly targeted treatment with respect to genotype or variant of PG. Current use of corticosteroids, immunosuppressants, and biologic therapies have shown moderate effectiveness, but a better understanding of PG may guide alternative therapies for cases refractory to these approaches. Furthermore, a standardized protocol would avoid missed diagnoses of PG. With criteria such as PARACELSUS and the criteria proposed by the Delphi exercise, we appear closer to a systematic diagnostic approach. Future studies should look toward increasing the number of individuals who achieve full healing without recurrence while reducing the potentially fatal complications that can arise from systemic PG.
